# Treatise on Cerebral Pathology; Containing Original Researches on the Structure, Functions, and Morbid Changes of the Brain, and the Therapeutical, Moral, and Hygienic Treatment of Its Diseases

**Published:** 1845-07

**Authors:** 


### Art. II.

*Traité de Pathologie cérébrale, ou des Maladies du Cerveau; nouvelles Recherches sur sa Structure, ses Fonctions, ses Altérations, et sur leur Traitement thérapeutique, moral et hygiènique.* Par Scipion Pinel, m.d. ancien médecin des aliénés de la Salpêtrière et de Bicêtre, &c.—*Paris,* 1844.

*Treatise on Cerebral Pathology; containing original Researches on the Structure, Functions, and Morbid Changes of the Brain, and the therapeutical, moral, and hygienic Treatment of its Diseases.* By Scipion Pinel, m.d., formerly Physician to the Lunatic Department of the Salpêtrière and Bicêtre Hospitals, &c. &c.—*Paris,* 1844. 8vo, pp. 564.

The treatise before us is elementary and practical; it is principally intended, as its author states, for the use of students and practitioners desirous of possessing a complete, though concise, history of the structure, functions, and diseases of the brain. It will be seen however, as we proceed in our analysis of the volume, that the author has not limited himself to a mere condensed account of the prevalent doctrines bearing upon his subject, but that in several important instances he has contributed the results of original investigations tending to throw considerable light upon certain obscure portions of cerebral pathology.

We have in various articles in this Journal made our readers so fully acquainted with the opinions of the authors upon whose works M. Pinel mainly founds his description of the anatomy and physiology of the brain, that we shall pass over the sections of his work devoted to these subjects. We are the more ready to adopt this course, as in point of fact the acquaintance of M. Pinel with the existing micrology of the organ is any thing but perfect; and we shall besides by such omission be enabled to devote greater space to the more attractive divisions of his volume.

Turning then at once to the pathology of the brain, we find M. Pinel commencing with disorders of intellect. These disorders he divides into states of exaltation; of depression; of total abolition. Under the first head he comprises—acute delirium, mania or maniacal furor, monomania bearing upon ideas, as distinguished from monomania bearing upon desires; and, lastly, cerebral hallucinations, in contradistinction to illusions of the senses. To the second head, that of depressed or lowered activity, we find referred—chronic delirium, melancholia (hypemania), stupor or cerebral œdema, common dementia. To the third class belong imbecility and idiocy. We shall not make any attempt at a complete sketch of the author's account of these different morbid states, but simply dwell upon such points under each head as particularly attract our attention.

*Acute delirium.* The clinical lectures of Dupuytren contain, as is well known, among others of their graphic delineations, a picture of the acute delirium which, in certain cases, supervenes soon after the receipt of some serious injury, as a fracture, dislocation, &c.; or, it may be, after the individual has simply undergone some trifling operation. This species of delirium, which the celebrated surgeon described under the title of nervous, under the presumption that it arose independently of actual morbid change in the cerebral substance, M. Pinel on the contrary regards as constituting the most characteristic symptom of the outset of acute inflammation of the periphery of the brain,—in a word of acute encephalitis, and of encephalitis so rapid in its course that it proves fatal, before it has run through its ordinary phases. But inasmuch as these phases are not anatomically exhibited, the chain of evidence that encephalitis is always actually present in such cases must, we contend, be regarded as defective.

M. Pinel has remarked, as others have done before him, that acute diseases of the lungs are less prone to give rise to sympathetic delirium than those of other organs; but he attempts no explanation of the fact.

Contrasting the effects of opium and belladonna on the brain, the author states that whereas (in these countries at least) the former acts rather by stupefying the individual than by exciting a particular flow of ideas, the latter invariably gives rise to the most curious illusions of sense, and a sort of forced gaiety. This special character in the delirium following excessive doses of belladonna is considered to be explained by the action of the drug on the optic thalami.

When the irritation productive of acute delirium is "direct, make free use," says M. Pinel, "of derivatives of all kinds, and of certain sedatives, but in spite of appearances, be chary of bleeding from the arm; venesection in the majority of cases converts acute delirium into chronic mania."

*Acute mania or maniacal furor.* M. Pinel holds tha tan attentive observer will always succeed in discovering in certain premonitory symptoms the proof of a "period of incubation," rarely occurring in cases of ordinary mental alienation. An illustration will explain the writer's notion. A poor clerk in a shop, generally of melancholy disposition, saw some omnibuses belonging to a newly-started company passing in the street, and was immediately seized with the idea of establishing a similar concern himself. From that moment sleep deserted him; his nights were spent in revolving plans in his head; his days in laying these before all the moneyed individuals of Paris. When the success of his schemes was made matter of question, his rage was unbounded. In the course of a few days a fit of the most violent mania prostrated the poor speculator. Now cases such as this might be cited in hundreds; we ourselves have known two within the circle of our own acquaintance during the last few years. No doubt then can be entertained of the accuracy of the description; but we doubt whether such intellectual demonstrations as those described can be regarded as evidence of the existence of the *period of incubation* of insanity, understanding this phrase in the ordinary sense. And for this reason: the period of incubation of any disease being once set in, the supervention of the disease itself in mild or severe form is a necessary sequence. Now it is certain that such speculative vagaries, as have been referred to, seize upon thousands of individuals who never exhibit distinct symptoms of mental alienation. The state of mind in question we regard as one which may or may not, by the excited state of cerebral circulation it maintains, produce actual mania; in fact as an exciting cause and not a part of this affection. But this view of the condition of things would not lead us to watch an iota less carefully, than M. Pinel recommends, persons exhibiting the phenomena of this "period of incubation."

On the other hand we do not mean to question the reality of a period of incubation in certain other cases, where clear and obvious exciting causes are deficient. When individuals exhibit repugnance for their ordinary occupations, avoid acquaintances for whose society they had previously displayed particular fondness, pass their time in ruminations unconnected with existing circumstances and for which no motive, intelligible to other persons, can be assigned; and when, joined with their perverted state of intellect, cephalalgia, congestion of the brain, feverishness, disturbance of digestion, depraved appetite, loss of flesh and other functional disorders are observed, a state exists which may be fairly considered to announce incubation of mania. These phenomena may exist for months, for upwards of a year, before the actual outbreak of maniacal furor.

The author describes the phenomena of invasion, of the period of excitement, the convalescence and recovery from mania, with graphic conciseness. He observes that although the doctrine of crises, as taught by the older observers, cannot at the present day be admitted, yet that the occurrence of critical phenomena, especially in connexion with maniacal paroxysms, cannot be questioned. He affirms also, as a matter of constant observation, that patients whose recovery has been marked by critical evacuations from the kidneys, skin, intestines, &c., are much less liable than others to relapse.

The termination of mania is sometimes of a most disastrous kind. Maniacal patients not very unfrequently die *suddenly*, after exhibiting a state of furious agitation for a period varying from two or three days to three weeks. According to M. S. Pinel these patients are the victims "not, as is even still said, of nervous apoplexy, but of the extreme degree of development of violent cerebral congestion, of per-acute encephalitis, suddenly arrested during its period of greatest intensity." In another class of cases, the termination of the malady, though equally fatal, is less abrupt. The maniac after months, or it may be years, of violent excitement becomes suddenly quiet and silent; a few days after this change, decomposition of the features takes place, the skin becomes dull and yellow coloured, the eyes lustreless and fixed; the whole system appears deeply affected. Death follows in a few days, preceded by stupor and paralysis. In such instances the state of congestion is found to have passed on to actual softening, or even purulent infiltration.

The connexion sought to be established thus between mania and encephalitis is obviously of importance; nine cases are related in its illustration. In the first of these an old woman, after exhibiting a state of furor for six months, became suddenly quiet and died in two days. General vascularity of the hemispheres, especially of the gray substance, which M. Pinel presumes to have existed without intermission for six months, appeared to explain the maniacal furor; partial encephalitis (red softening) to account for the sudden cessation of life. The "cerebral phlogosis suddenly passed here into the inflammatory state, just as pulmonary catarrh may become in the course of a few hours a true pneumonia." Now the evidence that in cases like the present the congestion discovered in the brain has existed unchanged for months, or years, is to our minds quite unsatisfactory. The illustration given of the conversion of congestion into inflammation is peculiarly unhappy; we have never seen, and do not believe in, the passage of catarrh into pneumonia.

In others of the cases reported by M. Pinel, various chronic lesions of the membranes exhibited themselves, and are presumed to have been the cause of persistent furor, a proposition which it would perhaps be difficult to contest,—although a fact forgotten by M. S. Pinel in the ardour of his descriptions, namely, that all the chronic morbid changes observed by him are met with in persons who have never been maniacal, is not very easily reconciled with his doctrine.

Viewing the matter in its connexion with pure morbid anatomy, and perhaps even with pathology, hypertrophy and eburnation of the cranial bones, is a condition of no mean interest. M. S. Pinel, commenting on one of the cases referred to in which this condition existed, considers it explained by congestion of the brain frequently recurring, causing consecutive disease of the pia mater and arachnoid, and eventually of the dura mater; the latter membrane having contracted adhesions with the skull is further supposed to have produced an "afflux of abnormal nutrition" to the bones, and so augmented their density and bulk. Facile enough is the explanation; but what of the cases (and they are neither few nor far between) in which eburniform hypertrophy of the skull exists perfectly independently of thickening or unusual adhesions of the dura mater, and, vice versâ, of those where the alterations of the meninges reach their extreme degree unattended with modified nutrition of the bones?

The afflux of blood required to produce "irritation capable of causing permanent delirium" eventually produces "a special decomposition" of the gray substance; this "decomposition" is signified by the apparent division of the substance in question into three layers: "1. The first or internal of three layers, that contiguous to the white substance, retains its grayish hue in very much the natural state. 2. The second layer, of greater thickness, and of bright or violet red colour, appears to be formed solely of vessels loaded with blood: in this stratum I place the seat of all maniacal symptoms, and of all states of morbid excitement of the intellectual faculties, when it presents a very bright red colour, and is increased in consistence. The degree of consistence, however, varies a little, with the greater or less advancement, and the more or less acute course of the disease; at an early period it is greater, towards the close less marked. 3. The third layer, much thinner, is pale and whitish; this is the albuminous exudation, which we found so distinctly marked in some cases, but which is in all distinguishable from the middle stratum by its grayish appearance." We have transcribed this account, though but little disposed to place unlimited confidence in its accuracy; we have examined some subjects cut off by encephalitis, and have not noticed this species of stratification, though not unaware that statements of its existence had been put forward.

*Acute monomania.* Acute monomania, no matter what be the nature of the idea on which it turns, is clearly connected with ordinary mania in consequence of the state of continual agitation of the sufferer, his outcries, and paroxysms of rage and fury. The violence of his fury is rendered greater from his being perfectly convinced of the reality of his chimeras, and from possessing the faculty of reasoning perfectly on the subject. Monomaniacs are among the most dangerous and intractable inmates of an establishment, says M. Pinel, and even their occasional fits of tranquillity are always to be looked upon with suspicion.

The suicide of the *suicidal monomaniac* is distinguished from that committed under the ordinary instigations to the deed by its being invariably perpetrated in a particular way. The monomaniac determines upon some system or mode of destroying himself, and nothing will divert him from its adoption; "the true suicide on the contrary avails himself," says M. Pinel, "of any expedient which chance may supply." This latter statement does not appear to be perfectly correct if received literally, though, no doubt, the distinction sought to be established by M. Pinel is in the main real. It does not appear to be correct, because we know it was shown numerically in the remarkable work of M. Guerry (Sur la Statistique morale de la France,) that the mode of destruction adopted by suicides varied with their sex, age, social position, &c., was consequently to a certain extent regulated by these, and could not be regarded as altogether a matter of chance. It may be urged, no doubt, that the conditions of age, sex, &c., referred to will place individuals in positions more likely to supply this or that ready means of self-destruction, and there would be some validity in the objection, but not sufficient to warrant the exclusiveness of M. Pinel's proposition.

The terms *hallucination* and *illusion* have been used by some writers as synonymous, by others in different senses. By hallucinations the author understands "certain sensations which perceived by the lunatic, appear to be excited in his brain through simple excitement of the organ, and without the actual presence of the objects which these sensations represent. Dreams are an example of hallucinations furnished in the healthy order of things. The term illusion is reserved by M. Pinel for diseased states of the senses and of sensibility. In hallucinations the brain is the only part actively engaged; in illusions on the contrary, impressions made on the organs of sense reach the brain in a perverted condition. Numerous remarkable examples, presenting however nothing new, are related of hallucinations of various kinds, and cases added exhibiting the transition from the state of hallucination to that of chronic mania. Indeed mental alienation might, in certain points of view, be looked upon as a series of hallucinations, deriving from these its most characteristic signs. In hypochondria cerebral hallucinations and illusions of sense coexist. By the way we may mention that M. Pinel defining hypochondria with many other writers as a perversion of the instinct of self-preservation and of love of life, takes umbrage at the term (which it must be confessed is sufficiently absurd,) and proposes the word *biophobia* should be adopted in its room. But biophobia can only be interpreted fear of life (like hydrophobia, photophobia, &c.,) and not, as M. Pinel's notion would require, fear for or concerning life; the word employed should signify fear of death.

The description of *ecstacy* is good, but requires no analysis, as it is almost verbatim derived from Andral and Calmeil.

In enumerating the causes of *chronic delirium,* M. Pinel alludes to the supposed influence of the moon in inducing paroxysms. "Daily experience at the Salpêtrière and at Bicêtre exhibits, in a collection of upwards of 3000 patients, the absolute nullity of the alleged influence of the moon. It may however be said that, during certain nights, lunatics may be more than usually agitated in consequence of the unusual light entering their rooms, and the figures produced on the walls and windows." This seems a plausible way of accounting for the marvels recorded concerning the influence of the moon on the insane.

Among the weakened conditions of the intellectual faculties, one of the most curious and interesting is that state of intellectual and moral annihilation, described by Esquirol under the title of *acute dementia.* This state differs from ordinary dementia in that it almost always affects young persons, and terminates rather rapidly in recovery. Georget described marked cases of it; and M. Etoc Demazy was the first to connect the symptomatic phenomena with serous infiltration of the cerebral substance—œdema of the brain. M. S. Pinel presented a special memoir on the subject to the Academy of Medicine in 1840, and submits the substance of this to the readers of his present work. As this affection is less known in its anatomical relations than it should be, we shall give a pretty full analysis of the author's descriptions.

The dura mater and arachnoid are generally natural in appearance but raised up by subjacent serosity. The pia mater is thickened, of pale pinkish hue, exhibits a varicose appearance, or sometimes seems studded with granulations or albuminous concretions; it is infiltrated with serosity of darker colour, and less viscidity than natural. The upper and lateral convolutions of the brain are flattened and pressed against each other; the appearance of the anfractuosities is almost lost; the gray substance loses its natural colour, becomes tumid and spongy, yet rarely loses its consistence; the proper structure of the part is infiltrated with serous fluid, and the vessels even contain this in abundance instead of blood; the white substance is similarly infiltrated, but to a less extent; its vessels likewise contain serous fluid, as those of the gray matter. The seat of this œdema is remarkable; in eight out of nine cases it was limited to the superior regions of the brain, either lateral, anterior, or posterior; in the ninth, the cavities, both internal and external, of the encephalic system were filled with fluid. In no instance did the liquid exhibit itself at the base of the organ. This affection of the brain generally coexists, or is complicated, with œdema of other parts of the body.

M. S. Pinel inquires whether the serous exhalation is produced through obliterations of the veins, which have in consequence lost their faculty of absorbing, or whether the irritation of the brain which produced delirium at one time has become transferred into a "secretive irritation which produces abolition of intellect." In replying, the author observes, first, that there is a particular tendency in these patients to œdema of various parts of the body. Now, in individuals possessed of this tendency, "it is conceivable," he continues, "the brain being in a state of irritation already, and its faculties excited above par, that this cerebral affection shall act in its turn upon the pia mater, increase the activity of its exhaling function, and eventually spread to it the state of morbid excitement affecting its own substance. The pia mater then becomes red and thickened; its vessels undergo dilatation; it secretes a greater quantity of serosity than natural, and absorption ceasing to hold the healthy relation to secretion, an unusual accumulation of serosity follows between the membranes and brain, just as occurs in the serous cavities. A new species of compression then acts on the brain, which changes the maniacal state to one of apparent calm, and which, making rapid progress, eventually blunts completely the faculties of intellect, locomotion, and sensibility."

Be this, or be it not, the real connexion of the anatomical changes, the symptoms of the affection are clearly defined. Those depending on alterations of sensibility require separate consideration, when this disease commences, and when it is confirmed; but the difference is one of degree and not of nature. At first various portions of the skin become more or less insensible; the fingers and other parts may also become the seats of various modified states of sensibility; the patient may feel a sensation of cold, &c., in these regions. In the most advanced stage the insensibility is such that the skin may be burned, or a seton applied, &c., without the least consciousness being exhibited on the part of the patient; the mucous surfaces are likewise perfectly insensible. The external senses only, says the author, are implicated; thus the conjunctiva is perfectly insensible, but vision excellent.

In respect of movement, the characteristic phenomenon of œdema of the brain is a state of numbness and unwillingness to move—a sort of *station fixe* of the limbs. This differs from paralysis completely; the patient can move, but it requires a violent effort of the will to produce movement. The author thinks the condition connected with cataleptic rigidity, and considers it worth serious inquiry whether the latter extraordinary condition be not the result of compression of the brain, generally or partially, by serous effusion.

The most violent delirium and maniacal agitation are suddenly exchanged in these cases for a state of perfect tranquillity and apparent rationality; the patient suffers the strangest hallucinations. Memory and the faculty of attention first become disturbed; the ideas confused and vague; speech slow, laborious, and impossible. At a more advanced period the intelligence is totally annihilated. But there is a very remarkable feature in the mental phenomena of the disease; the patients retain consciousness of their condition, for, on recovery, (which frequently takes place,) they affirm that they were perfectly aware of their position, but felt neither the strength nor the will, nor even the desire, to cause its cessation.

Cerebral œdema signifies its approach by cephalalgia and a feeling of constriction round the head; the organic functions suffer too; hunger, thirst cease to be felt comparatively; the excretions are obstructed; the pulsations of the heart fall to forty, or even thirty in a minute.

The author considers it readily conceivable that an affection which is "like an asphyxia of the intellect, although extremely serious, should nevertheless rarely prove fatal." Whether very readily intelligible or not, the fact is as stated. Out of one hundred and twenty-five insane subjects M. Etoc Demazy speaks of only five very obvious fatal cases; an experience of nine years furnished the author with but five others. Nevertheless acute dementia is sufficiently common; but it almost always terminates by recovery, after an ordinary duration of a few months.

*Simple common dementia,* that state of general weakening of the moral and intellectual faculties which constitutes the closing phasis of all cases of incurable delirium, M. S. Pinel attempts also to connect with a special condition of the cerebral substance. This is a peculiar kind of induration; the vessels disappear, the medullary substance acquires a dull whiteness, becomes hard, resisting, and fibrous. This induration is also, according to the author, frequently observed in epileptic subjects. He is, however, far from regarding it (daily experience might be cited to prove his error, if he did,) as the sole anatomical alteration of the brain met with in persons dying in a state of dementia; he simply maintains that the condition in question exists sometimes and had been but little observed.

The author passes to the subject of *idiocy* and *imbecility*. He admits three "perfectly distinct varieties" of idiocy—amentia (abrutissement); stupidity (stupidité), and silliness (bêtise); these varieties appear to us nothing more than degrees of a state fundamentally the same; one of the cases given shows indeed the transition of one into the other. Idiocy is congenital; imbecility acquired.

In *amentia* the total existence of the patient seems limited to digestion and respiration. Neither hunger nor thirst are felt, the individual remains in the spot and position in which accident has placed him. In *stupidity* the idiot has some consciousness of his wants; he cries out when hungry, and will move to look after his food. Some of those belonging to this category are in a state of ceaseless motion, motion without an object; they pass days or years in balancing the body backwards and forwards: they sit huddled up together in a corner, and as M. Pinel observes, seem to have a sort of attraction for each other; an attraction of which their passion for masturbation is the cause. In a less marked degree of idiocy, silliness (bêtise), the individual is susceptible of the influence of education to a certain extent, he gives evidence of some slight intellect, and exhibits propensities; he understands questions having reference to his own wants; he moves with an object and according to directions given, and is consequently capable of being made useful in household concerns, &c.

Now these conditions can only be regarded as degrees of each other, as is shown by the following case, which possesses interest of no ordinary kind in several points of view. A girl, hydrocephalic from birth, was brought to the Salpêtrière at the age of 16, in a state of complete *amentia*, incapable of action or comprehension in any single way; her limbs were not larger than those of a child of six years old. But after some months of care on the part of a nurse who had conceived an affectionate sympathy for the poor being, she had made such progress as to be able to knit, and to articulate a few words and phrases. In the course of a year, she was able to hold a sort of imperfect conversation in ill-articulated words, upon numerous subjects connected with herself; here, however, the improvement appears to have become suspended. The poets have made much of the influence of love, whether sexual or of a purer kind, in developing the intellectual faculties; here is a case in point. The picture is a more pleasing one, but not more true to nature in its way, than the "comment l'esprit vient aux filles" of Lafontaine.

The fifth chapter introduces us to "*lesions or perversions of instincts.*" The first instinct considered is that of self-preservation, the first perversion of this, hypochondria. The original mode of regarding this malady, in respect of its organic cause, was as an affection primarily of the abdominal viscera, secondarily of the brain. Georget and others, on the contrary, started the idea that the brain was really the organ first affected, and the

4297

author gives in his adhesion to the same notion, while he attempts even a greater closeness of localization than those persons. According to M. S. Pinel, "the central parts of the brain are earliest attacked, become the seat of a slight change, which gives evidence of its existence by the first symptoms of hypochondria, such as disgust of life, and imaginary apprehensions regarding everything which concerns health. Subsequently the affection becomes more fully developed, extends wholly or in part to the ganglionic system, &c. . . . Hypochondria is hence in my opinion a neurosis, cerebral at first, eventually trisplanchnic. It must consist in an irritative alteration, of slight amount at first, but which may acquire a great degree of intensity, especially during paroxysms and violent fits of the disease." This may or may not be correct doctrine, but the author advances nothing in the form of demonstration, and in the next page appears, in speaking of hypochondria which follows excessive sexual indulgence, to place the genital organs in the position which the generality of writers accord to the digestive, in other words that which he maintains is always occupied by the brain.

Suicide, considered as the result of perversion of the *instinct,* and wholly distinct from intellectual defect, is spoken of as acute and chronic. The acute suicidal mania is that following the course of an acute disease, a state which may be likened to a febrile impulse, which lasts from one to three weeks, and ends favorably if the individual find it impossible, in consequence of close supervision, to execute his design. The various motives leading to the more ordinary species of chronic suicide, that in which the individual nurtures and matures his plans for months and years with deliberate obstinacy, until chance or the occurrence of long awaited circumstances enables him to perpetrate his design, are examined with philosophic spirit, and there is so much valuable truth, as it appears to us, in the following observations, that we cannot regard their transcription as a trouble.

"The mania of self-destruction is at the present day propagated with such facility through all classes of society, that its existence appears to form an element in the manners of the times and to claim recognition as a consequence of modern civilization. It has ceased to excite alarm, it scarcely even surprises. The news of death, by his own hand, of a friend or relation, is received as a subect for curiosity rather than affliction, and the act itself is forgotten in the anxiety jo inquire after the details of its accomplishment. Here is a serious evidence of the blunted state of feeling, which arises from habit. But it must be confessed this indifference is traceable to a more deeply-seated and more comprehensive cause, none other than the moral apathy of the age, an age without faith in itself, in art, in literature, destitute of elevating creed of any kind; indifference to day, to be followed to-morrow by decline and corruption! Have we actually reached that point of weariness, which seemed to seize on old Rome, when there was nothing left for her to conquer?

" In an age of such moral constitution as this, need one feel surprise that individuals destroy themselves, much as they live, without having too clear a notion of the why or the wherefore? Can one marvel that suicide, heralded, published, as it is, in the daily prints, shall in the end consider itself something of importance, and stalk forth unabashed, as assassination has done too, in all its hideousness, before an age that receives it with very peals of applause? The legislators of old days were wiser than our own; they knew how to punish, or at least to stigmatise, suicide, self-murder, as well as the murder of one's neighbour. In Athens it was decreed that the head of the suicide should be burned separately; Tarquin the elder refused such criminals burial; in the times of St. Louis their bodies were dragged on a hurdle through the streets; at the present day, Roman Catholic priests refuse the suicide burial, but party-spirit deprives the lesson of the instruction it would otherwise convey. For my own part I believe the time has come for thinking seriously of stigmatising self-destruction as a vile and senseless act. If nothing better can be done, let us imitate the king of Saxony, and consign the bodies of suicides to the anatomical theatres. This simple measure would have the effect of arresting almost all women and many men in their suicidal design. Let the legislature also prohibit publicity being given to acts of suicide and homicide."

It is no doubt true that these observations are more strictly applicable to the state of feeling existing in France, than in our own country, on the subject of suicide; but there is much which has reference to ourselves. If it be certain that in these islands there is less propensity than in France to the commission of the deed for the mere purpose of acquiring a few hours' posthumous notoriety; and if disappointed young ladies and gentlemen less frequently among us bolt the door, kindle their *boisseau* of charcoal, and die lovingly in reciprocal embrace, it is as unquestionable on the other hand that example exercises a most pernicious influence in England, and that the crime is constantly committed without even the semblance of an intelligible motive. Epidemic suicide is even more common here than on the other side of the channel. As long as the pernicious system prevails among our jurymen, of shielding suicides from obloquy by their eternal and painfully absurd verdict of "temporary insanity," amelioration is neither to be expected nor imagined.

*Homicidal mania* presents two distinct forms. In one series of cases the murder is determined upon in consequence of false reasoning, or some senseless motive; in the other (and these cases are the more numerous), the intellect appears sound, the patient being led by a blind instinct, some indefinable feeling which drives him to the deed. Of both these varieties, striking illustrations are furnished either from the author's own experience or that of others. We shall content ourselves with noticing one example of the first species. An individual became suddenly impressed with the importance of purifying the human race by baptism of blood; he began in consequence by killing his children, and would have sacrificed his wife in the same manner, had she not escaped in time. Sixteen years later this same person, then confined at Bicêtre, succeeded in destroying two fellow-patients, a striking example of the pertinacity of an idea; his object was the same as before. Circumstances of recent occurrence in our own country, give all opinions bearing on the subject of such homicide peculiar interest, and we refer to the present chapter as to a source of sound instruction. The monstrous deeds which have been committed from time to time under the influence of this perversion either of intellect or of the affective faculties, and which have nevertheless been allowed to pass unpunished, excite a natural apprehension that real criminals may escape in company with those who cannot be esteemed accountable for their actions. The difficulty is increased by the fact that in certain cases of maniacal homicide, as of actual crime, there is premeditation and struggle with conscience, "there is consequently consciousness of murder." But this is too serious a question to be examined cursorily, and our want of space prevents us from entering upon a full inquiry into it.

But another class of persons, those who have been actually insane on other subjects for a greater or less period, are liable to be seized with the mania of homicide and destruction. In the insane, however, this "instinct of ferocity" is only an accidental complication implicating the affective faculties; whereas in those whom we have previously considered, it constitutes in itself the total malady. It is especially curious, and the remark has been made by numerous observers, that it is more common to find men who before their insanity had been distinguished for gentleness of temper and suavity of manners, exhibit, when insane, these destructive tendencies, than individuals otherwise characterized. The former class of persons, after recovery, dread their own character under certain circumstances, and fear at each moment that their determinations and acts may be somewhat influenced by the bias which exhibited itself so strongly during their attacks of madness: Esquirol relates the cases of two magistrates who having recovered from paroxysms of the kind, took a final resolution of never presiding in a criminal case, guided in this determination by the apprehension referred to.

The mania for destroying dwelling-houses, &c., by fire, is with as much difficulty explicable, as those varieties of perversion we have hitherto considered. The monomania is more frequent in some countries than in others, for example in Germany than in France. The late medical legist Marc supposed the fact (and more especially the greater frequency of perpetration of the deed by very young girls,) to be accounted for by the mode of life in Germany, and the circumstances attending the establishment of puberty, which there render nervous affections of a more extraordinary character and deeper die than elsewhere. Little importance can be attached to this hypothesis.

M. Pinel conceives that the title "*mania of temper,*" (reasoning mania of others,) may be applied "to that slight perversion of the instincts and affections, which makes an individual the scourge of all those with whom he is brought in contact, without his being, nevertheless, insane." He means those turbulent, indocile, reckless, ungovernable beings, constantly committing reprehensible acts, which they are always ready to justify by reasons, individuals who are a source of continual disquietude and anxiety to their relatives and friends. They know perfectly well what they do and say; the intellect is unaffected even, they defend themselves with clearness and force; in a word they labour under a perversion of instincts, under a generally increased intensity of bad propensities, but only to a degree which rarely amounts to actual insanity. This condition is one which requires to be studied and observed with the more care, that those affected with it are excessively cunning, and most ingenious in concealing their real state from persons appointed to inquire into their fitness for the management of their affairs.

A new chapter introduces us to the subject of "*lesions of voluntary or cerebral motion;*" they are considered under the heads of exaltation, depression, and intermittence.

Concerning the violent and powerful muscular action in maniacal and certain epileptic patients nothing new is said. Tetanus is said to be "the result of a still more marked" [than in mania and epilepsy, the influence is] "inflammation of the motor nervous centres. Subsultus tendinum, stiffness of the limbs or of certain muscles, convulsive motions giving a sort of quivering movement to these organs, are all symptoms which only occur towards the middle or close of cases of the most intense encephalitis; they are always bad signs, because they indicate most violent congestion of the brain, always followed by prompt disorganization and per-acute softening." In proof of all this we are simply told that upwards of fifty cases are to be found in the works of Rostan, Bouillaud and Andral, confirmative of its truth. Now there is no excuse for an author who makes such broad statements as these, and furnishes no precise evidence on this head: who will believe that the doctrine taught concerning the anatomical characters of tetanus is anything but a matter of closet speculation? Not those certainly who have either seen or read accounts of the post-mortem examination of individuals cut off during the existence of that morbid state. Every one of the symptoms M. Pinel enumerates as significant of extreme cerebral congestion, always followed by prompt disorganization, and per-acute softening, are witnessed in persons cut off by the French form of typhoid fever, (that which exists under his very eyes,) whose brain presents very trifling aberration only from the state of health.

For a greater or less period before their death, a certain proportion of insane individuals are afflicted with *general paralysis.* This affection was first made the subject of special study by M. Calmeil, and certain other French writers are quoted by M. Pinel as having investigated it at later periods. He makes no mention of Dr. Carswell, (which was of course to be expected, as he was not a countryman of the author's,) who has given a graphic delineation of the atrophy of the convolutions and accumulation of serosity in the meshes of the pia mater, which is certainly the anatomical condition discoverable in a certain number of these cases. It would appear, however, that such is not always the morbid change present, for the author, after a special experience in the matter (on the duration and closeness of which he dwells with complacency,) comes to the conclusion that under one vague generic title, several very different pathological states have been confounded,—states having each of them their characteristic course, symptoms, and morbid changes. These states are referrible to six perfectly distinct forms, according to M. S. Pinel's judgment; and as the subject is comparatively a new one, we shall give an outline of his statements concerning each.

The first form has not been hitherto described. It is that in which the general paralysis presents itself with all the symptoms of a most violent inflammation, running a rapid course of a few days or weeks, terminating fatally, and leaving obvious traces of inflammation in the centre or on the surface of the encephalon.

The second form is the first in a chronic state. The invasion is slow, sometimes imperceptible; the duration often more than six months or a year. The disease slowly disorganizes the cortical substance, especially its middle strata; traces of its presence are also very frequently found in the corpora striata, and the white substance surrounding the lateral ventricles.

The third form is marked by acute symptoms, which apparently acknowledge inflammation as their cause, andare yet the result of an "hypertrophy, an acute turgescence of the white substance of each hemisphere." The peripheric or cortical substance is perfectly healthy, but the convolutions are flattened against the skull. The anatomical characters of this hypertrophy, (especially as occurring in children, and under circumstances which render it liable to be confounded with hydrocephalus,) are well known; the new feature in its relations disclosed by M. S. Pinel, (in addition to its connexion with "paralysis of the insane,") is that of its coexistence with, and indeed production of a febrile state.

The fourth form is that of atrophy of the convolutions with effusion of serum, much more rarely of blood, within the meninges.

In the fifth form the paralysis affects an intermittent course. It recurs at intervals of six months or a year, disappearing completely, to return at fixed periods.

The sixth form appears to be founded rather on the curability of the disease, than on its peculiar anatomical or symptomatic characters. Recovery takes place in certain cases, it may be under the influence of treatment, or, as is infinitely more probable, from the peculiar character of the patient's constitution. The author has known patients, reduced to the last stage of marasmus, having lost the soft parts about the sacrum extensively by sloughing, and being at the point of death, nevertheless recover and even resume their station in society. Such fortunate events are, however, singularly rare.

Whatever be the nature of the cerebral alteration, the first symptoms noticed are difficulty of speech and deglutition, accompanied by disturbance of ideas. Such symptoms occurring in the insane, announce with greater certainty the outset of "paralytic encephalitis;" they will justify the physician in prognosticating a fatal event in individuals appearing at the moment possessed of full bodily health and vigour. At least so says M. Pinel in the paragraph now before us, but it is to be remembered that according to his own experience, recovery is not an actual impossibility. Shortly after the supervention of the above symptoms, weakness of the lower extremities sets in, with occasional spasmodic movements. Such may be considered the first stage of the disease.

In the second degree the symptoms become more marked; the upper extremities are seized with paralysis. The patients are unable to raise their arms; in some instances they clench convulsively whatever they hold in their hands. The sensibility of the body generally grows obtuse; the motions become involuntary and the paralysis extends to the muscles of the entire digestive system.

Incipient emaciation and the establishment of sloughing announce the third degree. The sensibility and intellectual faculties of the patient sink into a state of complete annihilation. Persistent contraction of the limbs, with partial convulsions, sets in. Various complications in the thoracic or abdominal viscera ensue, and the patients perish in the most complete state of marasmus, unless as sometimes happens they are cut off by sudden congestion during the second period of the disease.

The proper treatment of this terrible malady seems to be yet an enigma. M. S. Pinel, after having made trial of every mode of medication which reason points to, is obliged to confess his almost constant failure. He was not long in observing, however, that private patients occupying their own houses, surrounded with persons to administer to their wants, and, above all, to keep them in a state of cleanliness, had a better chance of recovery than the inmates of the wards at Bicêtre destined for the reception of the poor in such condition. Hygienic treatment, the circulation of pure air round the sufferer, &c., are consequently matters of primary consequence in the management of the affection.

The two chief therapeutical indications are to disgorge the brain of its excess of blood, and to support the strength. The first indication has been best fulfilled, according to M. Pinel's experience, by the daily abstraction of blood from the nucha by cupping, and this kept up for months together. But the quantity of blood he recommends to be taken is small certainly, otherwise this constant draining system would be unintelligible,—it is no more than half a table-spoonful. At the same time the patient's strength should be supported by tonic and substantial regimen. Certain it is that venesection, abstinence from food, and the antiphlogistic treatment, generally accelerates the progress and increases the severity of the disease,—a result of which reason would even à priori point out the likelihood.

Three causes of paralytic encephalitis have been ascertained by M. Pinel: these are, excessive indulgence in spirits; grief and physical misery; hereditary predisposition. In connexion with this etiology it may be stated that delirium tremens is, in the author's estimation, "nothing more than a first degree of paralytic encephalitis."

The account of *epilepsy* is graphic and concise, but not remarkable for its novelty. The author's experience leads him to doubt altogether the possibility of arresting a fit "by acting on the species of nervous current called the aura epileptica:" he has at least never seen anything demonstrating such possibility. As regards the nature and seat of the lesion giving rise to the malady, we find him discoursing as follows: "As in the case of general paralysis, so of epilepsy, the identity of seat, so vainly sought for, can only be explained by supposing the morbid state to implicate certain fasciculi, or fibres of certain of the more irritable portion of the encephalon. Hence that morbid state may be seated in the medulla, in the medulla oblongata, in the pons or cerebellum, or in the brain itself. It may depend upon a state of chronic inflammation, or simple chronic irritation, on atrophy or hypertrophy, on effusion or the presence of a tubercle, and, in a word, upon all lesions, trivial or serious, which are seated in the site of these motor and sensitive fasciculi or fibres."

*Hysteria* is defined to be in the female "an intermittent affection of motility, determined by morbid excitement of the genital system and its nervous plexuses, and characterized by sudden loss of consciousness, by general convulsions, and by the fact that after its cessation the patient is conscious of what occurred during the fit; the latter circumstance distinguishes hysteria completely from epilepsy."

In commenting upon the ordinary prescription in cases of confirmed hysteria, namely marriage, M. Pinel enters into a discussion concerning the primary seat, the fountain-head of the disease, and sneering at the well known crotchet of Georget (who placed the seat in question in the brain) recognises the genital organs as the primum mobile of all the phenomena: this is shown by his definition. Now as we confess ourselves to be of those who do not regard this question of seat as certainly determined in *all* cases, we shall state some of the arguments used by this author in support of the thesis he adopts. Here is a case. A lady's maid continued to follow an exemplary line of conduct till the age of twenty-six: at that period she began to experience certain nervous indefinable sensations, insomnia, tightness at the throat, suffocating feelings, general numbness, and fits of involuntary laughing and crying. Matters had come to this pass, when her services were required for the exhibition of an enema to a female fellow-servant; while thus employed she suddenly felt the genital organs inundated with fluid, lost consciousness for an hour, and on recovering found her propensities completely changed. She consulted several medical men, who were unanimous in counselling marriage. This being too slow a process, she applied at once to a male friend, who satisfied her desires; pregnancy followed. She produced abortion at four months and a half; horrified at all her criminalities, this victim of hysteria attempted suicide, failed, was brought to the Salpêtrière in a state of melancholia, and succeeded in hanging herself a month afterwards. Another still more convincing case follows,—convincing, we mean, as to the fact that in some cases the uterus is the source of the evil. But is it always so? Are there not cases in which slight symptoms similar to those of hysteria—hysterical in fact—are unconnected with uterine orgasm? we think there are. But we believe the question open to debate: prostitutes are not hysterical, and such symptoms as we allude to do not occur in women who have passed the age at which sexual desire commonly ceases.

Hysterical phenomena originate in the nerves and nervous plexuses connected with the genital organs; such is the author's notion, and it is only by admitting it, he conceives, that the occurrence of symptoms analogous to those of hysteria in the female can be understood iu the male. This appears *primâ faciè* a model of a *non sequitur*, nor does the legitimacy of the argument appear more obviously from the following case adduced in its illustration. A young man, aged twenty-two, was in the habit of feeling the globus hystericus ascend along the spine, and produce a choking sensation; he suffered from general nervous symptoms, with swelling of the abdomen, wandering of ideas, without convulsions; he was constantly on the point of losing consciousness, without ever actually doing so. The genital organs were but slightly developed, and he had never had intercourse with women; he had no beard. All this is very well; such cases occur from time to time; but, in the name of Zeno, what justifies M. Pinel's conclusion! "I have no doubt that there was here some lesion of the pelvic nerves or ganglia, analogous to that determining hysteria."

However, in justice to the author, as well as on account of the extreme interest attached to the circumstances, we must give an outline of another and infinitely more conclusive case. M. Régis, a captain in the army, of elevated stature, robust constitution, and largely developed abdominal system, was at Brussels in 1809, when he received a ball which passed obliquely from above downwards through the abdomen, entering above the nipple and escaping below the last lumbar vertebra. Shorly after (being made prisoner immediately), he had a very severe attack of intermittent fever; "which terminated in the sixth month by a singular crisis, a discharge of blood from the urethra of three days' duration. From this period he continued subject to this species of catamenial discharge every month. If exposed to fatigue, or to privation of food, or harassing attacks of the guerillas, the menstrual discharge became suppressed, and the captain suffered from the symptoms of amenorrhœa, such as colic pains, sensation of weight in the loins, heat and pain in the epigastrium, all of which continued until menstruation returned."

This condition of things grew worse and worse till the month of December, 1812. The symptoms had become complicated with convulsive attacks, recurring every month for two or three days before the discharge of blood. During these attacks the patient lost consciousness, had convulsive movements of the limbs, without stiffness, suffocation, delirium turning upon sabres, cannon, &c.; the respiration became slower, and vomiting sometimes occurred. These attacks lasted about two hours, recurred frequently in the course of the day, and only ceased on the appearance of the blood. Infusion of arnica and enemata of assafœtida appeared to have most effect in bringing on the discharge. What eventually became of the case is not mentioned; the patient had been under treatment for two years for these attacks, when he joined the Sardinian service, and appears to have been lost sight of.

Passing over a chapter of some length devoted to the consideration of "lesions of sensibility and the senses," which, though judicious and concisely complete, contains nothing specially worthy of notice, we reach the author's exposition of his views concerning the causes of cerebral diseases.

The predisposing causes are hereditary influence, the critical period, the progress of age, parturition, certain sorts of character, "according to Gall" certain conformations of brain, and the influence of public events.

Hereditary influence is powerful in the transmission of cerebral affections, and is more obvious in the highest classes of society than the middle and lower. The first families have "either been extinguished in this way, or have in the long run fallen into a state of inevitable intellectual degeneracy, because their position obliged them to intermarry constantly, and confine themselves within the narrow circle of a few privileged houses."

At the critical period the equilibrium of the functions is disturbed, and the desire of pleasing increases as the faculty of doing so fails; it is at this period that well-bred women take refuge in the practices of devotees, while the uneducated apply for consolation to the bottle. Of the former class of persons, many of the so-called religious meetings are in great measure composed. It is they that annually send forth ignorant men to waste funds (that might be employed in relieving the poignant misery of our alleys and lanes) in compelling the simple children of nature to embrace a faith to the precepts of which the conduct of their emissaries but too often furnishes the most flagrant contradiction. Were it not for "femmes passées," and hysterical young ladies, "qui se jettent dans la dévotion" in search of a strong sensation, some of our Halls might shut their doors, and probably some amount of the venom of sectarianism be transmuted into the milk of human kindness. Hysteria is in truth not a mere family or personal annoyance by its paroxysms and petty vagaries; it is often a political curse. Why is it that in so many remote nations of the earth the aboriginal spirit of charity and content is supplanted by civilized hatred and social bitterness? Simply because knots of women, old and young, whose natural feelings have been interfered with, and perverted by the laws of society or otherwise, find a vent for their passions in getting up meetings, applauding inflammatory harangues and "*ingenious devices*" from men whose zeal seems to be generally in the inverse ratio of their judgment. In alluding to these matters we are not wandering from the legitimate field of practical medicine: the physician can propose to himself no higher aim than the correction of moral evil by the prevention or cure of physical imperfection; and if, as we are persuaded, the giant pest to which we have made allusion be in great measure the offspring of hysteria, by improving the prophylaxis of this, he elevates himself to the rank of a political benefactor.

But of all the causes, the most powerful are those "which depend on the conformation itself of the brain, and on an abnormal predominance of certain of its parts over others. In spite of the numerous exceptions encountered in practice, the general result that the excess of certain masses of the brain in its anterior, lateral, superior and posterior regions, as observed in numbers of individuals of sound mind, leads to inferences concerning their intellectual and affective characters which are deficient neither in justness nor profundity." In this modified, and even timorous way, does M. S. Pinel profess his adhesion to the system of Gall. But we believe (that is we, the writer of this notice,) that such adhesion is all that experience justifies. Our conviction upon the philosophy of Gall is this. First, that *phrenology* properly so called, that is the science of mind, as taught by Gall, is founded in truth, but that it is matter of certainty the division of the intellectual faculties adopted by him is very imperfect, while none other, not open to objection, has been proposed since the announcement of his own. Secondly, that *craniology*, the doctrine which discovers a power in the organs of the various faculties of modifying the form of the skull, has also its foundation in truth. Thirdly, as respects *cranioscopy*, which is the art of practically applying craniology, we believe that it is sometimes successful, oftener fails, and, as conceived by its inventor, is destined for numerous and obvious reasons to remain, except as respects the grander divisions of the skull, an *impossible* art. Such an estimate of craniology can only be justified by experience,—to that experience we confidently appeal.

Respecting monomania of various kinds, as pleading against or in favour of Dr. Gall's doctrines, M. S. Pinel supplies no novel facts; but he insists on the importance of inquiring, in all available instances, into the evidence furnished by such cases.

In describing the "physical causes" of cerebral diseases, M. Pinel exhibits himself a disciple of a bygone school: "in describing these physical causes," he says, "we describe the diseases themselves, and the lesions on which they depend." Here the anatomical change is made the cause of the malady, itself constituted by a group of symptoms: this is neither consonant with existing doctrines, nor, we are persuaded, with sound pathology. The author attaches great importance, and probably not more than it deserves, to the particular manner in which he conceives the term, *seat* of the disease, should be understood in respect of cerebral affections.

It is not a particular part of the brain which will, in consequence of disease, produce such and such special symptoms; the very same mass of cerebral substance may give rise to very different functional effects, according as the intellectual, motor, or sensitive fibres composing it are specially altered. By attention to this suggestion (already made in an earlier paper) the *seat* of cerebral disorders will probably be eventually made matter of more ready determination than at present, when, as is notorious, such determination is, in the great majority of instances, impossible.

The first "physical causes" of cerebral maladies described by the author (they are described verbatim from Andral) are congestion and inflammation: states which we should regard as constituting the maladies themselves. There is nothing actually new in the author's comments on his extracts from Andral.

M. Pinel leads us to suppose that softening of the brain is particularly frequent among the aged inmates of the Salpêtrière during autumn. In them the disease "is rather a senile decomposition than an inflammatory process . . . . this cerebral softening is asthenic, it has not the strength to be inflammatory." No evidence is adduced in support of this notion, which appears perfectly deficient in clearness. Be this as it may, the symptomatic forms under which the disease may present itself, are, according to the author, as follows:

1. Sudden loss of consciousness, with simple paralysis.
2. Sudden loss of consciousness, with persistent contraction.
3. Sudden loss of consciousness, with general or partial convulsions.
4. Consciousness not lost, intellect somewhat blunted, sudden affection of movement.
5. Consciousness not lost, alteration of movement gradually effected.
6. Absence of usual symptoms.
7. Absence of symptoms of any kind.

The reader will do well to compare these groups of symptoms with the statements on the same subject given in our analysis of the elaborate work of Durand-Fardel. (Brit. & For. Med. Rev. vol. XVI, p. 1, 1843.)

It has been taught by several observers that hemorrhage into the brain sometimes, though very rarely, takes place in the direction of the strata of fibres of the organ, in such manner as to separate these fibres to a certain extent from each other without actually tearing the cerebral substance; and it has been inferred that such mode of hemorrhage produces a lesion more readily susceptible of cicatrization than the common lacerative variety. M. Pinel takes upon himself to deny the reality of the fact, and consequently of course the justness of the inference. Now we have not ourselves had actual *experience* of the justness of the inference; it would be inconceivably difficult to obtain it, as will become obvious on the least reflection. But the inference is a fair one, if the fact be real; and that it is real we have ourselves had distinct and satisfactory opportunity of observing.

That there are some cases of excessive rarity in which hemiplegia occurs on the same side of the body as the hemisphere implicated in hemorrhage, M. Pinel attempts to explain by supposing that some structural anomaly may have been present. "May it not sometimes happen," he urges, "that the usual decussation of the fibres of the corpora pyramidalis is in some individuals replaced by a state of simple juxta-position,—just as displacement of the heart occasionally occurs." The hypothesis is one which, at least, deserves to be put to the test of experience.

The author's observations upon "organic degeneration" of the brain are few and not remarkable always for accuracy. He says, for example, that cancer of the brain depends on a "general cancerous diathesis," the truth being that there is a tolerably considerable number of cases on record, in which that organ was the only one in the body affected.

The doctrines professed by the author in the earlier parts of his volume would lead us to anticipate that in its closing division on Treatment he would exhibit himself a firm stickler for a system of therapeutics based upon the organic changes presumed to be present in each instance of cerebral disease. And he realizes our anticipations. He appears to fancy that the present state of acquaintance with the anatomical changes attending various groups of symptoms is such, as to make it possible for the physician to guide his treatment towards the modification of the former, and not the mere management of the latter. The "medicine of symptoms" appears to him sadly contemptible in respect of maladies of the brain; and the "polypharmacy of the English" receives in particular more than perhaps its meed of castigation at the hands of this self-satisfied author of an impossible crotchet. Impossible, we mean, in the existing state of knowledge, impossible in respect of every organ of the frame, impossible *per eminentiam,* we should have presumed, in the case of the brain. What, M. Scipion Pinel directs his treatment to the removal of physical conditions or organic changes in epilepsy, for example! Would that we had his secret for their discovery; for though it is quite within the range of probability that the efforts to cure permanently and completely that malady might prove as abortive as according to our experience they do at present, even were an organic change proved to exist in all cases, and the mode of detecting its nature and degree during life made obvious, still it would be a source of satisfaction to the physician that his attempts were made, not as in the present state of things, but with a distinct object, and upon feasible grounds.

But (as was again to be anticipated,) the examination of M. Pinel's chapter on treatment, shows that his system could not be, even by himself, adhered to clinically. The details of the chapter gain in common sense, precisely what they lose in adherence to that system.

A vast number of cerebral affections present the symptomatic appearances of "inflammatory irritation." Here are comprised almost all such affections, indeed, at their outset; and here we meet two general indications of a precisely opposite character, depending on a state of hyperemia on the one hand, of anemia on the other. Fulness of the vessels is very frequent in acute affections of the brain, and is to be recognized by fulness and frequency of the pulse, and injection and a swollen condition of the superficial veins. "But," comments the author, "a remarkable fact which has repeatedly been proved by observation, is that this excess, often far from augmenting the acuteness of the cerebral symptoms, appears to calm and act as a sort of sedative upon them. Although the curative indication be to disgorge the sanguineous system, the indication must be followed up with extreme reserve. I have often seen women at the Salpêtrière, who had been treated elsewhere for temporary delirium, admitted in a state of furor, supervening after abundant loss of blood to which they had been submitted." True, and valuable as true; but if so, M. Pinel, what comes of the *anatomical* basis of treatment? And a little further on: "we often observe that the only effect of bloodletting is to diminish the state of hyperemia, and the intellectual disturbance continues in spite of it unaffected." Not a question of this; but might the fact not have led M. Pinel to reflect that there is something else beyond his state of hyperemia or anemia in the chain of morbid phenomena, and that at least as long as that something remains a secret, the notion of basing treatment upon lesions will continue a chimera.

M. Pinel's remarks concerning the use of the cold douche to the head in mania, deserve circulation; the shower-bath is but a variety of the douche, and the only variety almost used in private practice in this country. "If," he says, "some advantages have actually been obtained by the use of this agent, I must frankly avow for my own part, that its almost invariable effect is the precise contrary of that proposed. At the Salpêtrière the administration of the douche is a constant cause of exacerbation; the patients excite each other, vociferate, become riotous, or sometimes endure it with a forced calmness, which is not less injurious to them. ...... It is impossible to give it for any continuance to males, without causing general congestion and expression of the most agonizing suffering. . . . . Better effects are ensured by allowing cold water to fall in a very thin stream, or even guttatim on the patient's head after he has been bound down in a proper position in his bed. . . . The repeated application of ice to the head is also an excellent means of relieving irritation of the brain."

M. Pinel's ignorance respecting the state of medicine in England is remarkable even for a Frenchman: he is probably proud of his ignorance, and if so, he has much to be vain of. It is not true, we may venture to inform him, that it is the general habit of English physicians to salivate maniacal persons. Such obfuscated glimmerings as have reached this writer's intelligence concerning English doctrine and practice appear to be derived from tradition, hearsay, and a translation of Ellis on Insanity; all of them sources to which, we apprehend, the body of English physicians would object, as unlikely always to furnish the most satisfactory or accurate accounts in the world of what they profess, and what they do.

The affection, called paralytic encephalitis, of which we have very briefly described M. Pinel's pathology in a previous page, may be considered to be represented by four principal forms of morbid change: acute inflammation; chronic inflammation; hypertrophy; atrophy. Are these affections distinguishable during life inquires the systematist; and may this diagnosis be established with such surety as to permit the treatment to be directed to each one of them in particular, according to circumstances? No, responds the practitioner. And he responds wisely; but what comes of the pretension to treat lesions and not symptoms, of the philosophy that disdains effects and looks to causes? It remains, where it was conceived, in M. Pinel's easy chair, whence if it have ever travelled to the bed-side, we venture to affirm it can only have done so to the disadvantage of the patient, and the discomfiture of its author.

The symptoms of the affection are always identical in nature, "with the exception of inevitable differences in their course, their mode of invasion, and their severity,—all of which vary with predisposition of the individual." That is, they are identical in the present state of knowledge, but that they will always continue so, or that they are so in truth, is a matter of utter unlikelihood. When they have ceased to be so practically, M. S. Pinel may teach us how to treat lesions, meanwhile he contents himself with controlling symptoms, after the fashion (already referred to) of taking half a spoonful of blood by cupping from the nape of the neck every day for several successive months. At the same time he gives a weak infusion of arnica internally; and supports the patient with a nourishing and gently tonic diet. When the disease has advanced to the second stage, the great point is to prevent excoriations of the surface, by careful watching. In the advanced period of the affection, the two evils to be contended with are obstinate constipation or as unmanageable diarrhœa.

The indications in cerebral œdema are to free the brain by diuretics and revulsion. It is to be observed, that in this affection purgatives must be given in enormous doses to produce their usual effects; the author has administered twelve and fifteen drops of croton oil, without any other result than the production of ordinary evacuations. The same may be said of various systems of counter-irritation; they do not give rise to their ordinary painful effects, until some amount of improvement in the state of the patient becomes visible. Bloodletting increases the severity of the symptoms. Cold baths, and the application of cold water to the head, do not appear beneficial; but it is imagined that the affection is one in which sudden immersion in water (bains de surprise) would prove useful, by acting at once physically and morally, and "producing a salutary commotion." However, as the affection is rarely dangerous, and consists solely in a temporary suspension of the intellectual, sensitive, and motor functions, and as its duration may be diminished by revulsive and diuretic treatment, the author considers it much more prudent to limit ourselves to the latter plans.

If we were asked to state fairly our experience of the treatment of epilepsy, we should reply in the following terms, which we can scarcely doubt will accord with the opinions formed on the subject by the majority at least of our readers: that the malady may be rendered less distressing, that is by diminution of the violence and frequency of the paroxysms, under systems of treatment the most various, and some of them the most simple,—that an absolute cure can be *expected* from none, and is of infinitely rare occurrence under all circumstances. M. S. Pinel appears to have more confidence in belladonna than in any other drug; has tried indigo, without any other effect than that of dyeing the nails blue; has failed in producing any improvement with carbonic and hydrocyanic acids, the white oxide and the potassio-tartrate of antimony. The nitrate of silver "has often produced most serious effects on the stomach." M. S. Pinel has opened the body of an individual who had taken it for eighteen months, and found the stomach corroded in several places, and deprived of its mucous membrane at the fundus; whence he infers that nitrate of silver destroys the mucous membrane. Whether it do or do not, the conclusion is, on the evidence adduced, palpably defective; where is the proof that the state of the membrane described was not produced by the gastric juice?—the appearances were precisely those of post-mortem softening. This is not the only instance in which but moderate familiarity with morbid anatomy and the necessities of logical reasoning is exhibited by the author of this volume.

The chapter upon the moral treatment of insanity is judiciously written, but we do not find any precisely novel suggestions. Importance is attached to sea-voyages, and especially the continued sickness with which in some persons they are attended: one favorable case is referred to in support of this opinion. Under the head of hygienic treatment, the most fitting mode of organization of an establishment for the insane, the classification of patients, the important question of their regimen, and that of the employments of various kinds to which they should be daily set, are successively considered. These chapters, if condensed, would lose their value, and had therefore better be consulted in the original, by those whom they are likely particularly to interest.

On the whole, this volume may fairly claim a place on the shelves of the practitioner particularly devoting himself to mental diseases; had it less of pretension about it, it would probably be read with more advantage, and would certainly do more credit to its author.

---

